# Crosstalk between the gut microbiota and innate lymphoid cells in intestinal mucosal immunity

**DOI:** 10.3389/fimmu.2023.1171680

**Published:** 2023-05-25

**Authors:** Yuling Guo, Yujia Liu, Binqi Rui, Zengjie Lei, Xixi Ning, Yinhui Liu, Ming Li

**Affiliations:** Department of Microecology, College of Basic Medicine, Dalian Medical University, Dalian, China

**Keywords:** gut microbiota, innate lymphoid cells, lamina propria, diseases, intestinal mucosal immunity

## Abstract

The human gastrointestinal mucosa is colonized by thousands of microorganisms, which participate in a variety of physiological functions. Intestinal dysbiosis is closely associated with the pathogenesis of several human diseases. Innate lymphoid cells (ILCs), which include NK cells, ILC1s, ILC2s, ILC3s and LTi cells, are a type of innate immune cells. They are enriched in the mucosal tissues of the body, and have recently received extensive attention. The gut microbiota and its metabolites play important roles in various intestinal mucosal diseases, such as inflammatory bowel disease (IBD), allergic disease, and cancer. Therefore, studies on ILCs and their interaction with the gut microbiota have great clinical significance owing to their potential for identifying pharmacotherapy targets for multiple related diseases. This review expounds on the progress in research on ILCs differentiation and development, the biological functions of the intestinal microbiota, and its interaction with ILCs in disease conditions in order to provide novel ideas for disease treatment in the future.

## 1 Gut microbiota and intestinal mucosal immunity

A wide range of microorganisms colonize in the human intestinal mucosa, including fungi, viruses, and bacteria, which are together called the gut microbiota. The development of the intestinal microbiota during early life is a dynamic process that is influenced by various perinatal conditions, such as delivery method, feeding type, and lifestyle (1), and contributes to numerous physiological functions (2). The gut microbiome can affect host nutrient utilization *via* intestinal epithelial cells (IECs) and Paneth cells and even regulate immune cell proliferation and function (3, 4). The gut microbiota interacts with the immune system of the intestinal mucosa, generating a comprehensive intestinal defense mechanism. This interaction supports signal conduction pathways that regulate intestinal mucosal immune function, and the defense system jointly resist invasion by foreign pathogens both directly and indirectly (5). ILCs, a type of natural immune cells enriched in human mucosal tissue, have attracted extensive attention in recent years (6), as they play a critical role in regulating regional immunity and homeostasis.

### 1.1 Gut microbiota

The gut is the main site of nutrient and water absorption during digestion, and it constitutes a fundamental barrier against harmful substances and pathogens from the external environment. The gut microbiota plays a critical role in supporting intestinal barrier function (7). The humans gut contains hundreds of trillions of beneficial bacteria, representing a symbiotic relationship arising from millions of years of co-evolution. These bacteria make a significant contribution to human intestinal health (8), contributing to physiological functions such as carbohydrate digestion and fermentation, vitamin production (9), epithelial cell maturation (4, 10), angiogenesis (11), and lymphocyte development (12–14). The gut microbiota is also essential for protecting hosts from pathogenic infections. For example, the gut microbiota can limit the luminal colonization of intestinal bacterial pathogens through competition dietary nutrients (15). Gut commensal microbes also elicit protective immune responses against parasites *via* dendritic cells (DCs). In the absence of TLR11, immune responses to parasites is depend on the indirect stimulation of DCs provided by commensal microbes. Gut bacteria will activate MyD88 *via* TLR2, TLR4, and TLR9 to generate IL-12 which could response to the parasite (16). Furthermore, several studies have indicated that the intestinal microbiome is involved in the regulation of energy homeostasis, such as the synthesis of gut peptides (GLP-1, PYY) involved in energy homeostasis. High-fat diet induced obesity and metabolic disorders may associate to the innate immune system (2).

However, despite the symbiotic relationship between humans and their gut microbiota, the effects of these microbes are not always benign. When the balanced interrelationship between the host and the intestinal microbiome is destroyed, intestinal microbes may cause diseases (17, 18). For instance, *Enterococcus faecalis* is a gram-positive intestinal commensal bacterium, but it can invade mucosal tissues and opportunistically cause bacteremia and endocarditis (19). In addition, irritable bowel syndrome (IBS) had been linked to significant changes in the microbiota, specifically involving several groups of *Firmicutes* and *Proteobacteria* (20, 21). Lipopolysaccharide (LPS) derived from intestinal microbes has been recognized as one of the triggers for obesity and related metabolic (such as insulin resistance and type 2 diabetes) (22). Our understanding of the role microorganisms play in disease and health continues to widen, and even small disruptions have been found to break this symbiotic relationship and cause a microenvironmental imbalance.

Accumulating evidence demonstrates the close relationship between the gut microbiota and human diseases. For instance, various studies indicate that patients with IBD have an altered gut microbiome (23–26). Decreased concentrations of short-chain fatty acids (SCFAs) produced by gut microbes in IBD patients affect the differentiation and development of Treg cells and the growth of IECs (27), which are important for maintaining intestinal homeostasis. In individuals with food allergies, *Clostridium* causes the colonic lamina propria to produce a large amount of TGF-β and promotes Foxp3^+^Treg differentiation (28). Subsequently, the Foxp3^+^Treg cells inhibit inflammatory responses through TGF-β, IL-10, and IL-35, thereby maintaining oral tolerance to food (29) and alleviating this phenomenon, such as nausea, vomit, diarrhea, and pruritus. The microbiota also has a special significance in the context of cancer. The gut microbiota can regulate the immune system and thus produce anti-cancer effects. A study revealed a significant decrease in intestinal *Lactobacillus reuteri* and SCFAs, especially acetate, in mouse models of hepatocellular carcinoma (HCC). Moreover, it showed that the supplementation of *Lactobacillus reuteri* or transplantation with fecal bacteria from wild-type mice had significant anti-tumor effects in HCC mice (30).

### 1.2 Intestinal mucosal immunity

Multicellular organisms interact with their external environment at multiple niches, including the airway mucosa, oral mucosa, gastrointestinal and urogenital mucosa, and skin mucosa, which together comprise the mucosa-associated lymphoid tissue (MALT). The gut is an organ with the largest surface area and mucosal interface (31, 32), and it can continuously interact with dietary antigens and a variety of microorganisms. Therefore, the surface of the intestinal mucosa is a critical site for innate and adaptive immune regulation (33). MALT is a peripheral immune organ including gut-associated lymphoid tissue (GALT), nasal-associated lymphoid tissue, and bronchial-associated lymphoid tissue, and its functions are similar to those of the spleen and lymph nodes. GALT, one of the largest lymphoid organs, is mainly present in the intestinal tract and its surrounding tissues. Its surface area is 230-300 square meters and it represents the “first line of defense” of intestinal mucosal immunity. Histologically, GALTs include Peyer’s patches, crypt patches, isolated lymphoid follicles (ILFs), and mesenteric lymph nodes (MLNs) (34, 35). GALT also includes non-immune cells such as M cells (36) and immune cells such as helper T (Th) cells, Treg cells, cytotoxic T lymphocytes, and IgA-producing B cells. ILCs, phagocytes, DCs and macrophages (37) are all mon-traditional lymphocytes. Numerous experimental studies have been that the intestinal microbiota is crucial for the development and maturation of GALTs (17). Both germ-free (GF) and specific-pathogen-free (SPF) mice exhibit impaired GALT development, which mainly manifests in crypt patches and ILFs (38, 39). However, if the intestinal microbial system of GF mice is reconstructed through microbiota transplantation, the mice can regenerate GALT (40) and their mucosal immune system is restored (41). In recent years, researchers have discovered new types of immune cells — different from traditional T and B lymphocytes — in the intestinal mucosal immune system. These cells, called ILCs, have become a new research hotspot (42). They are present in lymphoid and non-lymphoid organs and are enriched at the mucosal barriers, where they are exposed to allergens, commensal microbes, and pathogens (43).

## 2 ILCs

### 2.1 Differentiation and development of ILCs

From 2009 to 2010, lymphocyte-like non-T cells, non-B cells, and natural killer (NK) cells were described in several papers (44–47). The name “ILCs” to describe these cells was first proposed in 2013 (48). ILCs constitute a new and unique family of innate immune cells, including some well-known NK cells and lymphoid tissue-inducer cells and recently discovered non-cytotoxic ILC populations (49). ILCs exist in almost all organs and tissues (such as the lungs, liver, stomach, intestine, pancreatic islets, adipose tissue, spleen, and lymph nodes) (50–52), but they are enriched in mucosal tissues and play a key role in maintaining homeostasis and tissue repair.

ILCs are derived from common lymphoid progenitors (CLPs) in the bone marrow of central immune organs (53) and display a lymphoid morphology. The differentiation of ILCs is regulated by a variety of transcription factors, including inhibitor of DNA binding 2 (ID2) and nuclear factor, interleukin 3 regulated factor (Nfil3), and GATA binding factor 3 (GATA3). CLPs differentiate into common helper innate lymphoid progenitors (CHILPs) under the action of ID2, Nfil3, and GATA3, and then divided into innate lymphoid cells progenitors (ILCPs) under the action of promyelocytic leukemia zinc finger (PLZF) (54, 55). Driven by transcription factors such as T-bet, GATA3 and RORγt, ILCPs are differentiated into ILC1s, ILC2s and ILC3s (56, 57) respectively. CHILPs are differentiated into lymphoid tissue inducer progenitors (LTiPs) and further differentiated into LTi cells. In humans and mice, NK cells develop from CILPs through NK cell precursors (NKP), while ILC1s develop from CILPs through ILCPs (55). ILCs can mature without the recombination machinery and do not express a TCR. Activation signals in the immediate environment can control the ILCs function and producing copious cytokine (58). In the same way, ILCs play important protective roles in the early immune response against cellular transformation and infection, especially in epithelial barrier surfaces (59). Meanwhile, inflammatory diseases may contribute to ILCs dysfunction (60, 61).

ILCs are innate immune cells with adaptive immune function (55). Their subsets have the typical morphological characteristics of lymphocytes (58) but lack antigen-specific receptors. Therefore, they are distinct from adaptive lymphocytes (such as T cells and B cells) (62). However, it has been highlighted that ILCs are “mirror cells” of CD4 Th cells, ILC1s being the innate counterpart of Th1s, ILC2s of Th2s and ILC3s/ILCPs of Th17s. According to research finding, distinct expression of long noncoding RNAs (lncRNAs) in innate vs. adaptive cells, arguing for a potential role of lncRNA in shaping human ILCs biology. ILCs are unique in the dynamics, fine-tuning, and spatial organization of immune responses, rather than redundant genetic organization (63). Like Th cells, ILCs do not undergo gene rearrangement (64), subsets of ILCs are phenotypically unstable and can alter their own functions and associated phenotypes in response to microenvironmental changes (65). This phenomenon is called plasticity.

Plasticity begins before the terminal differentiation stage of ILCs subpopulations in mice (66). The *in vitro* transformation of ILC3s, ILC2s, and ILC1s is reversible. For example, human ILC3s are converted into ILC1s under the action of IL-12. Meanwhile, this process can be reversed by IL-1β and IL-23 and accelerated by the vitamin A metabolite retinoic acid (67). ILC2s can be transformed into ILC1s under the influence of IL-1β and IL-12 *in vitro*, but IL-4 can reverse this process (68). Additionally, NK cells can be unidirectionally converted into ILC1s during a *Toxoplasma gondii* infection (69). In principle, the forced expression of EOMES under the action of the Tbx21 promoter enables the transformation of mouse ILC1s into NK cells, although this process does not occur under physiological conditions (70). After *Salmonella enterica subsp.* infection, under the action of IL-12, NKp46^+^ILC3s downregulate the expression of RORγt and transdifferentiate into ILC1s (71, 72), contributing to defense against bacterial infection. However, the accumulation of ILC1s and IL-17-producing ILC3s has been observed in the inflammatory tissues of patients with Crohn’s disease (73, 74), accompanied by a reduction in the number of IL-22-producing ILC3s. These changes in ILCs composition have been linked to disease severity (75). Furthermore, the conversion of NKp44^-^ILC3s to NKp44^+^ILC3s has been observed in the inflamed skin of patients with psoriasis, leading to an increase in the number of NKp44^+^ ILC3s, which is correlated with psoriasis severity (76, 77). This suggests that the plasticity-dependent transformation of ILCs is required for their anti-infection and autoimmune functions.

### 2.2 Classification and functional characteristics of ILCs

#### 2.2.1 NK Cells and ILC1s

Similar to cytotoxic CD8^+^ T cells, NK cells have cytotoxic functions (78). They are capable of eliminating a variety of cancer cells, including some forms of leukemia (79). In addition, NK cells have proven to be useful in the treatment of cancer, especially during bone marrow transplantation (80). While ILC1s are usually non-cytotoxic or weakly cytotoxic, like Th1 cells (74, 81, 82), they represent the first line of defense against viral infections and some bacterial pathogens. NK cells and ILC1s depend on the key transcription factor T-bet. NK cells and ILC1s differ slightly in function, for example, NK cells are cytotoxic cells that express perforin, while ILC1s only expresses low levels of perforin. In mice, ILC1s can be detected before birth, while NK cells appear two to three weeks after birth (83). They are further differences in the production and dependence of transcription factors. In mice, ILC1s are strictly dependent on T-bet, while NK cells can also exist in T-bet deficient hosts (84). In addition, NK cells require the transcription factor Eomes, while ILCls can develop in the absence of Eomes. Therefore, Eomes expression is often used as a marker for NK cells, although it can be expressed in a certain proportion of ILC1s (55). However, once stimulated by IL-12, IL-15, or IL-18, they produce tumor necrosis factor (TNF) and interferon-γ (IFN-γ) and thus provide protective immunity against viruses (85), intracellular bacteria (81) and parasites (72).

#### 2.2.2 ILC2s

As for Th2 cells, RAR-related orphan receptors α (RORα), T cell factor 1 (TCF1), and GATA-3 (55, 86–88) are guide the development and maintenance of ILC2s and the production of type 2 cytokines. In mice, the markers of ILC2s are usually CD25, KLRG1, ICOS or ST2, while in humans, ILC2s is recognized by the expression of CD161, ST2, and CRTH2 (89). Following stimulation by IL-25, IL-33, or thymic stromal lymphopoietin (TSLP), ILC2s produce IL-5, IL-13, IL-6, and IL-9 (86, 89–92) and play a role in defense against nematode and other worm infections (93, 94). ILC2s can also produce amphiregulin to restore the integrity of the epithelial barrier after tissue damage (95). In addition, after acute infection with the influenza virus, ILC2s produce the epidermal growth factor family member amphiregulin and thereby promote the healing of respiratory tissue (96). In addition, after acute infection with the influenza virus, ILC2s produce the epidermal growth factor family member amphiregulin and thereby promote the healing of respiratory tissue (86, 89). Moreover, recent studies have revealed that ILC2s are present in the skin and may contribute to skin inflammation (e.g., atopic dermatitis [AD]) (97, 98).

#### 2.2.3 ILC3s and LTi cells

Similar to Th17 and Th22 cells (55), ILC3s require the transcription factors ROR gamma-t (RORγt) and aryl hydrocarbon receptor (AHR) for their differentiation, development, and function (99). ILC3s can secrete cytokines such as IL-17A, IL-22, TNF, and granulocyte macrophage colony-stimulating factor (GM-CSF) (51, 100, 101). As a result, they can help the body resist extracellular bacteria, promote the development and repair of lymphatic tissues (102), and maintaining intestinal homeostasis (101, 103). IL-22 can promote the production of mucin by IECs and maintain the proliferation of intestinal crypt stem cells (104), thereby protecting the integrity of the intestinal epithelial barrier (105). IL-17 can protect IECs by enhancing the synthesis of tight junction proteins and thus maintain intestinal homeostasis (106). In addition, IL-17 can also activate secretory factors and growth factors in IECs and endothelial cells to induce neutrophil differentiation, thus influencing the immune response to inflammation (107). ILC3s generate immune tolerance to the microbiota and protect gut health *via* antigen-specific RORγt^+^ Treg cells and Th17 cells (108). They maintain intestinal microecological homeostasis, and their down-regulation can lead to colitis (109, 110). In addition, ILC3s are also engaged in the defense against *Salmonella typhi* (111), *Candida albicans* (112), *Streptococcus pneumoniae* (113), and they prevent symbiotic translocation and systemic inflammation (96). Moreover, some studies have found that ILC3s can promote the regeneration of the inflamed intestine (114) and regulate allergic airway hyperresponsiveness in the lungs (115), This indicates that ILC3s are involved in the maintenance of tissue homeostasis during inflammation or injury (116).

**Figure 1:**
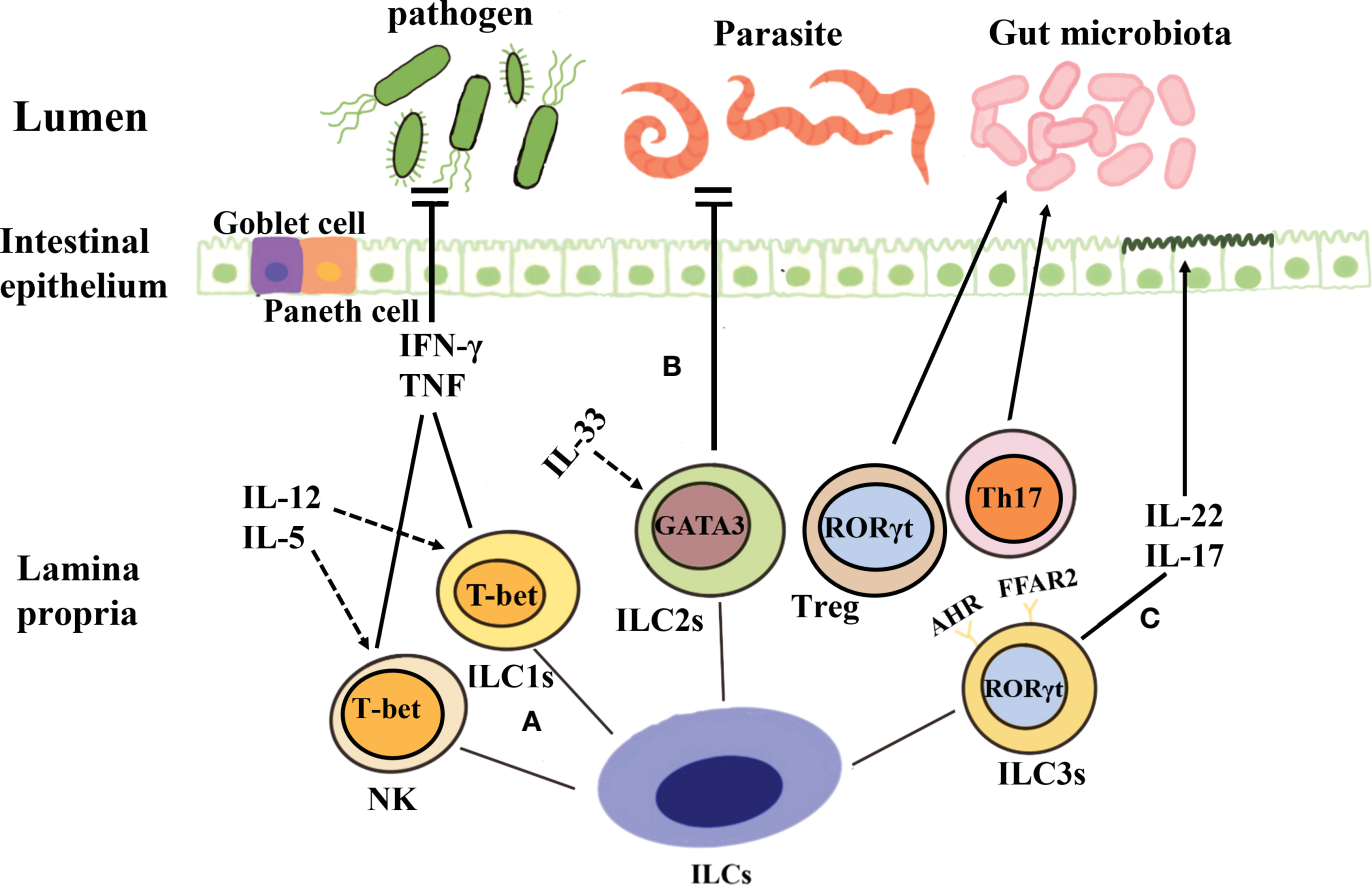
Classification and function of ILCs. **(A)** Both NK cells and ILC1s depend on the key transcription factor T-bet and secrete tumor necrosis factor and IFN-γ when they are stimulated by IL-12, IL-5, etc., to produce protective immunity against viruses, intracellular bacteria, and parasites. **(B)** The development of ILC2s is dependent on the transcription factor GATA3, and these cells produce cytokines such as IL-5 and IL-13 under the action of IL-33 and thus contribute to the prevention of parasitic infection. **(C)** ILC3s are dependent on the transcription factor ROR-γ, and they can secrete IL-17 and IL-22, protect intestinal mucosal epithelial cells, maintain intestinal homeostasis, and aid mucosal repair during the acute inflammatory stage. Further, ILC3s can also establish immune tolerance to the microbiota and promote intestinal health through antigen-specific RORγt^+^Treg and Th17 cells.

LTi cells were first described in 1997 (117), They express c-kit and CCR6 and are dependent on the transcription factor RORγt (55). Under the influence of TNF-α and lymphokine β, LTi cells play a key role in the formation of secondary lymph nodes and Peyer’s plaques during embryonic development (118, 119). In adult mice, LTi cells, B cells, and DCs together constitute ILFs (120). In recent years, several studies have shown that LTi cells express IL-22 through NF-κB and Toll-like receptor 2 signaling (121). In addition, LTi cells also secrete IL-17A and IL-22, which enable the protection of the gastrointestinal tract from pathogens (122) (**Figure 1**, **Table 1**).

**Table 1:** A summary of the main function of different innate immune subsets and their phenotypes related.

Cell subset	Cytokine	Main function	Crosstalk between ILCs and gut microbiome	Reference
NK cells	IFN-γ, TNF	Anti-virus Anti-tumor	Reduced cytotoxic activity of NK cells in GF mice Increased permeability and translocation of commensal bacteria in human intestinal epithelial cells	(123) (124)
ILC1s	IFN-γ, TNF	Anti-virus Anti-bacteria Anti-protozoa	Low amounts of ILC1s in fetal gut Inhibition of ILC3s to ILC1s conversion plasticity in the absence of symbionts Dysregulation of *Helicobacter typhlonius* and colitis in T-bet-deficient mice	(67, 73) (125) (126)
ILC2s	IL-5, IL-6, IL-9, IL-13, Areg, IL-33	Anti-parasite Asthma and allergic diseases Metabolic homeostasis Maintaining microecological Balance	Increased ILC2s counts in the small intestine of GF mice Butyrate-induced inhibition of IL-5 and IL-13 production in ILC2s *via* HDACs Reduced bacterial translocation and inflammation *via* Areg after DSS induction IL-33 can activate intestinal ILC2s to produce more Areg and maintain the composition of intestinal microbiota and microecological balance	(127) (128) (129) (130) (131)
ILC3s	IL-17A, IL-22, GM-CSF	Intestinal homeostasis Anti-bacteria	Differentiation of ILC3s and production of IL-22 dependent on gut bacteria Microbiota inhibit IL-22 secretion by ILC3s Microbiota induces ILC3s to produce IL-22 to strengthen the intestinal barrier Tryptophan-induced to increase IL-22 production by ILC3s Bacterial translocation and systemic inflammation in the absence of ILC3s	(132) (133) (28, 134) (135) (96)
LTi cells	IL-17A, IL-22	Induces the formation of secondary lymphoid tissue	Peptidoglycan from Gram-negative bacteria enables LTi cells to accumulate in intestinal crypt patches Impaired maturation of isolated lymphoid follicles in GF mice	(38) (38, 136)

## 3 Interaction between ILCs and the gut microbiome

In healthy people, the mucosal immune system shows a symbiotic association with the gut microbiome. Most lymphocytes in the intestinal lamina propria are only separated from the commensal microbiome by a single layer of epithelial cells. The stable relationship between the intestinal epithelial barrier and symbiotic bacteria depends on the immune system. Further, the commensal microorganisms also have profound effects on homeostasis and the development of the mammalian immune system. As an important part of the immune system, ILCs play a vital role in maintaining this symbiotic equilibrium. Thus, they can affect microorganisms at both the individual and community levels (**Table 1**).

### 3.1 Intestinal microbiome regulates ILCs

#### 3.1.1 Normal gut microbiota regulates ILCs

The intestinal microbiota does not appear to be necessary for the development of most ILC groups. However, signals from commensal bacteria can promote good expression of cytokines in the intestine and significantly affect the function of ILCs. The differentiation and development of NK cells do not require the role of the microbiota, but in the absence of symbiotic, the function of NK cells changes. The cytotoxic activity of NK cells and cytokine production are both reduced in GF or antibiotics in the treatment of mice (123). The reason is perhaps that the type 1 interferon produced by dendritic cells and macrophages promotes the production of IL-15 (137, 138), which in turn further promotes the maturation of NK cells. Therefore, the symbiotic microbiota controls the generation of NK cells indirectly (139, 140). Researchers have found that the number of ILC1s in the fetal intestinal is extremely low (67, 73), indicating that the development of ILC1s is dependent on commensal microorganisms. At the same time, in the absence of symbionts, the plasticity of ILC3s to ILC1s transformation was inhibited (125). There is no significant difference in quantity and proportion in ILC2s and their markers (IL-25 and IL-33 receptors, IL-7Rα and T1/ST2 and c-kit receptors) in GF and SPF mice (95). This indicates that the development of ILC2s does not require microbiota (139). However, lack of commensal bacteria significantly increased the proportion of ILC2s in the intestine (127). Research has shown that the microbiota regulates the function of ILC2s in the intestine by promoting the release of IL-25, thereby improving the intestinal barrier mediated by ILC2s (133, 141). In recent years, research has found that LTi cells also express the Toll-like receptor 2, and can express IL-22 through NF-κB signaling pathway (121). This means that LTi cells may directly perceive the cell wall components of Gram-positive bacteria. The peptidoglycan of gram-negative bacteria enables LTi cells to accumulate in the lamina propria of intestinal crypt patches (39). Maturation of ILFs is impaired in the intestines of GF mice, which indicates that some functions of LTi-like RORγt^+^ ILCs are disrupted (39, 142). Intestinal microbiota promotes IL-1β production by myeloid cells in tissues of mice, IL-1β stimulates ILC3s to generate IL-2, thereby promoting the generation of Treg and the intestinal tolerance to dietary antigens (143). CD11c^+^ myeloid dendritic cells produce IL - 1β and IL - 23 in response to intestinal microbiota, and participate in the production of IL22 of ILC3s (144). Stimulating dendritic cells TLR5 with bacterial protein flagellin promotes the production of IL-23, thereby leading the production of IL-22 through ILC3s (145).

#### 3.1.2 Pathogenic bacteria regulate ILCs

Due to the proximity of ILCs to the mucosal surface, ILCs are susceptible to various symbiotic and pathogenic bacteria. ILCs can respond to a variety of pathogens to protect the host and maintain tissue integrity. For example, infection with *Clostridium Rodentiae* can increase the number of ILC1s in the small intestine (146), which may due to the plasticity of ILC1s and ILC3s in the intestine. Recent studies have shown that *Helicobacter pylori* infection can lead to an increase in ILC2s in gastric tissues of human and mice, accompanied by the production of IL-5 and an increase in the number of B cells (147, 148), it was found that the proportion of B cells significantly decreased in GF mice infected with *Helicobacter pylori* treated with anti-IL-5 neutralizing antibodies. In the large intestine of mice lacking adaptive immunity, the introduction of *Helicobacter species Helicobacter Apodemus* and *Helicobacter pylori* can activate ILCs and induce intestinal ecological disorders (149). However, *Helicobacter pylori* significantly inhibited T-bet expression, while T-bet negative ILC3s cells showed no significant changes, but their proliferation ability was significantly reduced (150). *Salmonella typhimurium* selectively enhanced ILC3s to produce IL-22 which could promote infection (151). In addition, *Salmonella typhimurium* can invade ILC3s and cause Caspase-1 mediated ILC3s pyroptosis (151). On the contrary, the loss of caspase-1 in ILC3s leads to an increase in ILC3s survival and IL-22 production, thereby enhancing *Salmonella typhimurium* infection in mice. In addition, *Salmonella* infection can induce NKp46^-^ILC3s to differentiate into ILC1s. Buonocore et al. (152) found that *Helicobacter hepatica* can induce IL-23 dependent colitis. Increased production of IL-17 and IFN-γ by ILC3s is associated with the development of colitis. *Bacillus anthracis* disrupts the function of ILC3s in the body through the deadly toxin of anthrax, resulting in a decrease in IL-22 produced in MAPK signaling pathway, leading to further imbalance of the gut microbiota (153). During *Citrobacter* infection, ILC3s can secrete lymphotoxins, which bind to LTβR on dendritic cells and trigger their production of IL-23. IL-23 can stimulate ILC3s to produce IL-22, thereby protecting the host (154). In addition, during the period of *Citrobacter* infection, the expression of GPR183 on LTi cells promotes the LTi cells migrated to Peyer’s patches and ILFs (155), and is also crucial for the protection against *Citrobacter* infection mediated by ILC3s (156). After infection with *Citrobacter*, the expression of STAT3 in ILC3s is enhanced and binds with IL-22 to exert anti-infective effects (157).

#### 3.1.3 Diet regulates ILCs *via* gut microbiota

More and more evidences suggest that ILCs are related to diet and its metabolites. The lack of dietary vitamin A is expected to result in an abnormal decrease in ILC3s, a severe deficiency in IL-22 production, and a greater susceptibility to gastrointestinal infection with *Citrobacter rodents* (158). Retinoic acid (RA), a vitamin A metabolite produced by dendritic cells in the intestinal lamina propria, promotes the expression of ILC1s and ILC3s gut homing receptors (159), and enhances ILC3s function (160) and the production of IL-22 by up-regulating RORγt (161). On the contrary, RA promotes ILC2s proliferation by raising IL-7Ra, and the amount of ILC3s in the intestine of vitamin A deficiency adult mice decrease. Increased ILC2s during vitamin A deficiency can enhance the efficiency of *worm* expulsion (158). Existing studies have shown that in the period of micronutrient deficiency, ILC2s is maintained through fatty acid metabolism, which can maintain the production of IL-13 (162). Thus, the dietary status of the host can alter the balance of the ILCs and selectively optimize the immune response, thereby altering the propensity to infection.

The main metabolites of undigested carbohydrates in the colon microbiota are SCFAs (including acetic acid, propionic acid, and butyric acid). SCFAs are not just the body’s energy source (163), but also promote immune dynamic balance through their interactions with host cells (164). For example, *Lactobacilli* can use tryptophan to increase IL-22 production in ILC3s (126). Ligands generated *via* tryptophan metabolism can activate AHR, thereby promoting ILC3s function (129, 165). SCFAs can mediate host defense against *Citrobacter rodent via* their receptor free fatty acid receptor 2 (FFAR2) (166, 167). In ILC3s specific knockout of FFAR2 mice, the number of CCR6^+^ILC3s and the production of IL-22 are decreased, that leading to a decrease in the production of mucin and antimicrobial peptides, exacerbating *Clostridium difficile* enteritis. Conversely, SCFAs or FFAR2 agonists fed to dextran sulfate sodium (DSS) induced colitis mice can increase the production of ILC3s and IL-22 in the mice colon, which has protective effects on intestinal injury (99, 167, 168). In GF or antibiotics treatment mice, the IL-22 producing ability of ILC3s was enhanced (114), on the contrary, adding butyrate to ILC3s will limit the production of IL22 *in vitro* experiments (169). In addition, some SCFAs can stimulate AKT - STAT3 and ERK - STAT3 signaling pathways to induce IL - 22 producing of ILC3s (167). The IL-22 produced not only maintains the richness of the microbial community, but also resists fungal colonization such as *Candida albicans*, thereby preserving the mucosa from inflammation (135).

### 3.2 ILCs affect the intestinal microbiome

#### 3.2.1 NK cells and ILC1s affect gut microbiota

NK cells and ILC1s have been demonstrated to increase cell permeability and the translocation of symbiotic bacteria in human intestinal epithelial monolayers (124). CNK cells mainly circulate in the blood or reside in the bone marrow and lymphatic organs. The production of large amounts of IL-12 after infection with *Toxoplasma gondii* can induce the transition of NK cells to the ILC1s-like phenotype (Eomes CD49a Ly6C). The gut microbiota interacts with NK cells, such as feeding mice a high-salt diet can increase the abundance of *Bifidobacterium* in the intestine, resulting in increased intestinal permeability and localization of *Bifidobacterium* within the tumor, thus enhancing NK cells function and tumor regression, and injecting *Bifidobacterium* into tumors can activate NK cells and inhibit tumor growth (170). Hypercaloric diets overactivate the intestinal immune system, disrupts the microbiome and epithelial cell function. A new reserach found that in the hypercaloric diets fed mice, ILC1s depletion is also associated with increases in *Akkermansia muciniphila* and decreases in *Bilophila* spp. The expansion of pro-inflammatory macrophages and ILC2s needs ILC1s, and the ILC1s depletion induced the ILC3s-IL-22 pathway, consequently, increase production of mucin, antimicrobial peptides, and further affect the gut microbiota (171).

#### 3.2.2 ILC2s affect gut microbiota

ILC2s are a class of innate immune cells involved in IL-33 signaling, especially promoting type 2 immune response (172). ILCs are enhanced on the barrier surface and have been shown to be an integral part of mucosal repair in the case of infection (173). IL-33 activates ILC2s in the intestinal, producing Areg more significantly than ILC2s in other mucosal sites (130). IL-33-deficient mice were ecologically disequilibrated or had higher concentrations of pro-inflammatory bacteria constituting their microbiome. Specifically, IL-33-deficient mice had more *Segmental filamentous bacteria* (SFB), higher concentrations of pro-inflammatory bacteria were also found in a mouse model of inflammatory bowel disease, especially increased *Akkermansia muciniphila*, which can degrade mucus (131). The possible cause may be a lack of IL-33 fails to activate the IL-33/ILC2s pathway, which changes the structure of gut microbiota and increases the abundance of pro-inflammatory bacteria. When the epithelial barrier is destroyed, the AREG produced by ILC2s can reduce DSS-induced intestinal injury and cause the translocation of intestinal symbiotic bacteria to peripheral organs (129). Just like the gut microbiome directs the migration of ILC2s from the gut to the lungs *via* the gut-lung axis. When *Proteobacteria* abundance increases in the gut during abdominal infection, IL-33 production is stimulated, IL-33-CXCL16 signaling promotes natural ILC2s accumulation in the lung to protect the lung from infection. When ILC2s accumulated to a certain extent, the composition of gut microbiota changed (174).

#### 3.2.3 ILC3s and LTi cells affect gut microbiota

ILC3s do not directly affect the microbiota, but indirectly shape the microbiota by altering epithelial cell function or through other immune or non-immune cells functional characteristics, thereby affecting its composition. Blocking IL-22 or reducing ILCs can lead to the growth of *Alcaligenes* (usually present in Peyer’s patches and MLNs) in the liver and spleen, causing systemic inflammation (96). IL-22 expressed by ILC3s is necessary for fucose on the surface of IECs. This mechanism helps prevent bacterial transmission and is related to the prevention of intestinal *Salmonella* infection (111). In addition, IL-22 produced by ILC3s can promote the production of antimicrobial peptides and mucus in epithelial cells (175, 176), resulting in the host defense against pathogens such as *Citrobacter rodentiae* (46, 175). If IL-22 is absent, it will lead to a significant reduction in antimicrobial peptides and mucus, thereby affecting the composition of the intestinal microbiota, such as reducing the proportion of *Lactobacilli* (177). The Reg3 family of antimicrobial lectins produced by IL-22, such as Reg3γ and Reg3β, it can maintain spatial separation between host tissues and commensals, limit the total number of gut bacteria, especially those associated with the mucosa, and prevent microorganisms from translocating through the epithelial barrier and spreading to the MLNs and liver, thereby limiting inflammation (178–180). Studies have shown that mice who lacking IL-17A and IL-17F developed spontaneous infection with *Staphylococcus aureus*, while IL-22^-/-^ mice showed increased colonization of *Staphylococcus aureus* and decreased expression of antibacterial proteins by 10%. The production of IL-17 and IL-22 is related to ILC3s. This indicates that IL-17A, IL-17F, and IL-22 can regulate the colonization of *Staphylococcus aureus* (181, 182), and *Candida albicans* (112, 183, 184). Programmed death-1(PD-1) is substantially expressed in colonic LTi cells, anti-PD-1 immunotherapy in cancer patients can leads to immune-related adverse events such as colitis. During DSS-induced colitis, LTi cell activation is accompanied by increased PD-1 expression, at the same time, a dramatic reduction of *Lactobacillales* can be detected (185). Another research reported that the proliferation of LTi can be resistant to *Clostridium difficile* infection (186). In summary, the interaction between gut microbiota and ILCs plays a crucial role in maintaining the homeostasis of the gut environment.

## 4 Interaction between ILCs and symbiotic bacteria under disease

The mutualism between the microbes living in the intestinal tract of mammals and their hosts is critical for the development and maintenance of the immune system. Many inflammatory diseases and chronic infections in humans are related to changes in the composition or colonization of symbiotic bacteria, which lead to dysfunctional symbiotic relationships. In the last few years, studies have demonstrated the interaction between ILCs and the gut microbiota. The intestinal microbiota regulates the number of ILCs, thus affecting their development and function. ILCs are also essential for regulating the balance of the gut microbiota, suggesting that ILCs and the intestinal microbiota may play critical roles in disease pathogenesis. For example, ILCs participate in the pathogenesis of microbe-related diseases such as IBD, allergic diseases, cancer, and parasitic infection.

### 4.1 Inflammatory bowel disease

IBD is a major intestinal pathology, and it mainly includes Crohn’s disease (CD) and ulcerative colitis (UC) (142, 187, 188). IBD is simply an intestinal mucosal abnormality caused by environmental, genomic, microbial, and immunological factors (189). The proportion of NKP44^+^ILC3s in inflammatory tissue is decreased in IBD patients, and the proportions of ILC1s and ILC2s and levels of GM-CSF are all increased in IBD patients (190, 191), and it correlates with disease severity. IL-33 can promote the activation of ILC2s in the colon and induce type 2 immune repair pathways to avoid toxin mediated epithelial damage (192). In the absence of specific T-bet in ILCs, IL-7Rα enhanced the proliferation and accumulation of ILCs, which can promote the development of ILC2s in order to mediates type 2 intestinal immune response. Abnormal activation of the immune system could be prevented in this process, and the host would be protected from colitis (193).

ILC3s play a crucial role in regulating the stability of the intestinal mucosa. Several studies show that segmented filamentous bacteria and invasive *Escherichia coli* adhering to the intestinal mucosa of IBD patients can enhance the production of IL-22 by ILC3s (194, 195). In CX3CR1^+^ mononuclear phagocytes, these factors can also induce the expression of TNF-like ligand 1A, which plays a role in mucosal repair during acute inflammation (196). Free fatty acid receptor 2 (FFAR2) is the receptor for SCFAs and is highly expressed on the surface of colonic ILC3s. It can upregulate the expression of RORγt, act on IL-22 to promote intestinal IEC repair, regulate the secretion of antimicrobial peptides in the small intestinal mucosa, prevent invasion by pathogenic bacteria, and inhibit inflammation (105, 197). Natural FFAR2 ligands such as acetate and propionate as well as FFAR2 agonists help in increasing the production of ILC3s and IL-22 in the mouse colon and can protect against DSS-induced colonic injury and *C. rodentium* infection (167). AHR, as an important transcription factor regulating ILC3s, is expressed on the surface of ILC3s. AHR-deficient mice show increased susceptibility to colitis and *Citrobacter rodentium* infection due to impaired ILC3s and low IL-22 in the intestine (198–200) and the decreased number of intraepithelial lymphocytes (201). *Lactobacillus reuteri* can prevent DSS-induced colitis in mice *via* tryptophan metabolites such as indole 3-aldehyde, an AHR ligand that activates AHR and enhances IL-22 production (202–205).

**Figure 2:**
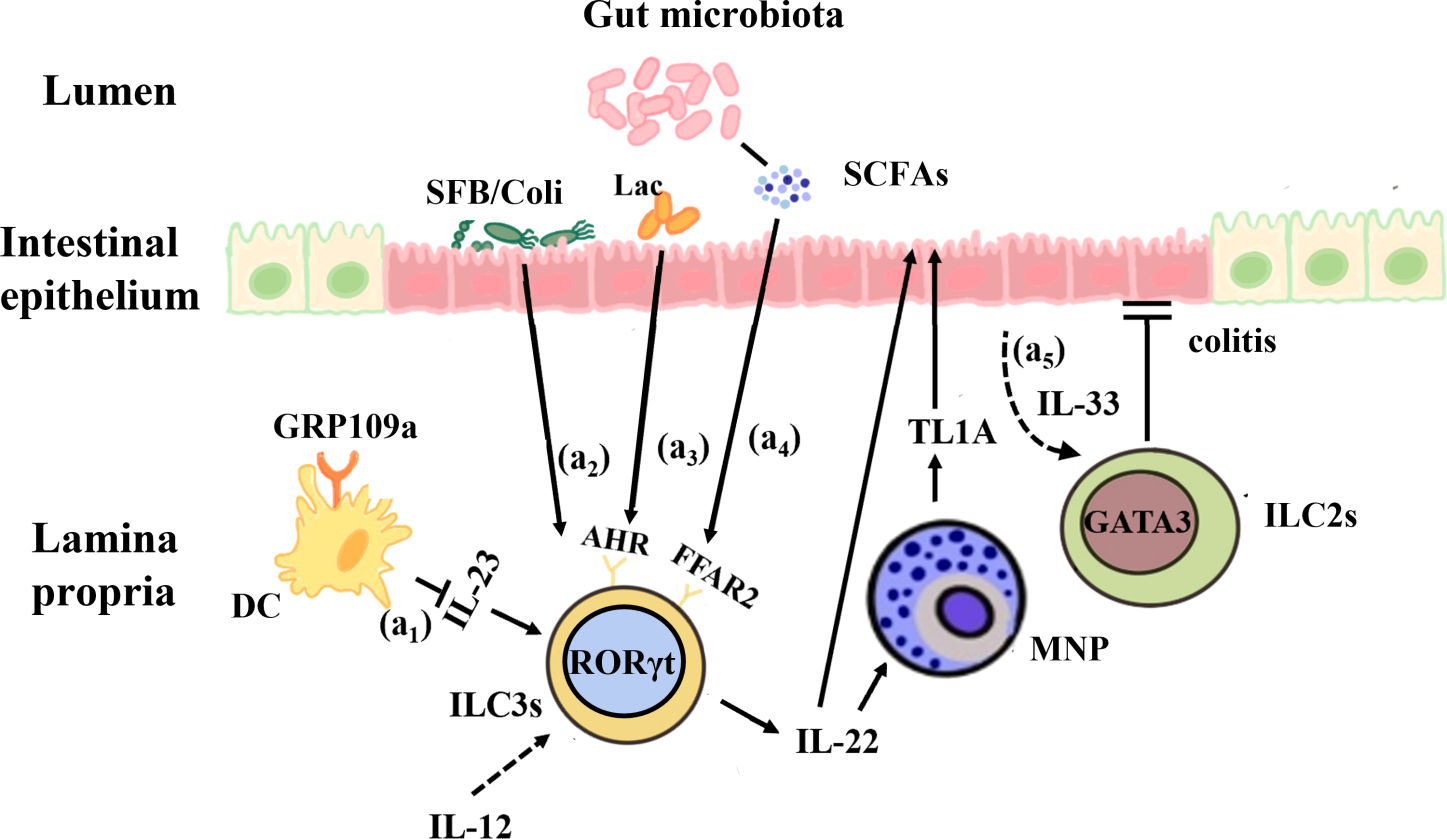
Interactions between the gut microbiota and ILCs in IBD. (a1) GPR109a regulates intestinal homeostasis by inhibiting IL-23 production in intestinal dendritic cells in order to inhibit ILC3s. (a2) Segmented filamentous bacteria (SFB) and invasive *E. coli* adhered to the intestinal mucosa of IBD patients can enhance the production of IL-22 by ILC3s and promote the expression of MNP-induced TL1A, which contributes to mucosal repair during the acute inflammatory stage. (a3) *Lactobacillus reuteri* can prevent DSS-induced colitis in mice by using tryptophan metabolites to activate AHR and enhance IL-22 production. (a4) The microbial metabolites of SCFAs, whose receptor FFAR2 is highly expressed on the surface of colonic ILC3s, act on IL-22 to promote intestinal epithelial cells and mucosal repair. (a5) IL-33 promotes ILC2s activation and protects against *Clostridium* -induced colitis.

However, ILC3s — which produce IL-22 — can also lead to the development of acute congenital colitis in mice (206). Compared with healthy controls, patients with mild to moderate IBD show higher levels of IL-22. IL-22 levels are also significantly elevated in CD patients (207). This increase has been positively linked to disease activity (208). An initial study, it was found that the elevated secretion of pro-inflammatory cytokine IL-17 and IFN-γ from NCR^-^ILC3s in the colon of a *Helicobacter hepaticus-*induced IBD model, could further promote intestinal inflammation. However, when these cells were depleted, the disease activity was attenuated (152). In addition, Gpr109a^-/-^Rag1^-/-^ mice were found to develop spontaneous colitis, and compared with Rag1^-/-^mice, they showed higher IL-17 production in intestinal ILC3s. This indicates that GPR109a can suppress ILC3s by inhibiting the microbiota-induced production of IL-23, thereby regulating intestinal homeostasis (209). Immune checkpoint blockade (ICB) immunotherapy is a common clinical anti-cancer therapy, but it can also cause serious side-effects. The most common side-effect is immune checkpoint blockade associated colitis (ICB-associated colitis). Supplementation with *Lactobacillus reuteri* can alter the composition of the intestinal microbiota, reduce the number of ILC3s, and then inhibit the development of colitis, thereby improving the weight loss and inflammatory state induced by ICB treatment. The pro-inflammatory effect of ILC3s may be due to their abnormal activation, leading to the overproduction of IL-22 and IL-17, which induce the production of neutrophil chemokines by IECs, as a consequence, exacerbates the inflammatory response (210). When the intestinal microbiota is disrupted, Th17 cells secrete a large amount of IL-22, causing IL-22 production to far exceed normal levels. This promotes the proliferation of colonic epithelial cells, stimulates abnormal mucosal dysplasia, and induces colitis (211, 212) (**Figure 2**).

### 4.2 Food allergy

Atopic diseases, including atopic dermatitis, food allergy (FA), and environmental allergy, have become important public health problems worldwide (213). FA is an antigen-specific biological response resulting from exposure to orally ingested food or food-derived components. While anaphylaxis, mast cell degranulation can be mediated by IgE, the reaction that can lead to death. Previous studies have demonstrated that changes in the structure of the gut microbiota can lead to a partial loss of homeostasis, disrupting the Th1/Th2 balance and biasing it toward the Th2 response, thus inducing allergic reactions (214, 215). Intestinal bacteria, especially *Clostridium*, play a major role in regulating mucosal immunity and allergic diseases. Stefka et al. found that *Clostridium* colonization induces IL-22 production in ILC3s and T cells in the intestinal lamina propria, which enhances epithelial barrier function *via* effects on goblet cells and Paneth cells that increase the production of mucus and antimicrobial peptides (28, 134). IL-22 inhibits the pathway mediating the entry of oral dietary antigens into the systemic circulation and contributes to the avoidance of food allergy. In addition, the SCFAs produced by *Clostridium* can induce the production of Tregs in the colon, promote the integrity of intestinal epithelium, alter the composition of intestinal microbiota, and improve allergy symptoms in mouse models (216, 217). Research from Ha-Jung Kim’s group shows that in the early-life of mice, intestinal microbiota can play a critical role in the development of AD by regulating the CD4^+^IL17^+^T cell/CD4^+^FOXP3^+^Treg cell balance, thereby affecting the levels of ILC3s in the gut mucosa *via* the modulation of SCFAs production (218).

However, ligands produced from tryptophan, a metabolite obtained from the diet and bacterial metabolism, interact with AHR on the surface of ILC3s. This can also stimulate the production of IL-22 to modulate the permeability of the epithelial barrier (135). If the intestinal mucosal barrier is damaged, intestinal permeability increases, harmful substances from the intestinal lumen, such as pathogenic microorganisms, antigens, and pro-inflammatory factors, enter the circulation. This can lead to food allergy symptoms. Intestinal microbes also can directly interact with macrophages, DCs, and ILCs to promote the production of defensins, thereby strengthening their barrier function and maintaining the mucosal immune balance (219).

The intestinal microbiota can regulate the intestine–skin axis *via* direct and indirect pathways. The changes in the composition and proportion of gut microbiota communities can cause skin barrier dysfunction and immune system disorders, which are the key pathophysiological mechanisms of AD development. Tryptophan is is a metabolite of the gut microbiota, it act as ligands that can activate the AHR on ILCs to induce IL-22 secretion, driving the release of antimicrobial peptides (AMPs) and providing protection from infections by pathogens (220). *Lactobacillus* and *Bifidobacterium* produce gamma-aminobutyric acid (GABA), which could be used by inhibitory interneurons as neurotransmitters to inhibit pruritogenic response neurons. Scratching also stimulates ascending nociceptive neurons that project to the supraspinal structures, which in turn connect directly or indirectly to descending modulatory pathways to inhibit pruritogenic response neurons, relieving itching (221).

**Figure 3:**
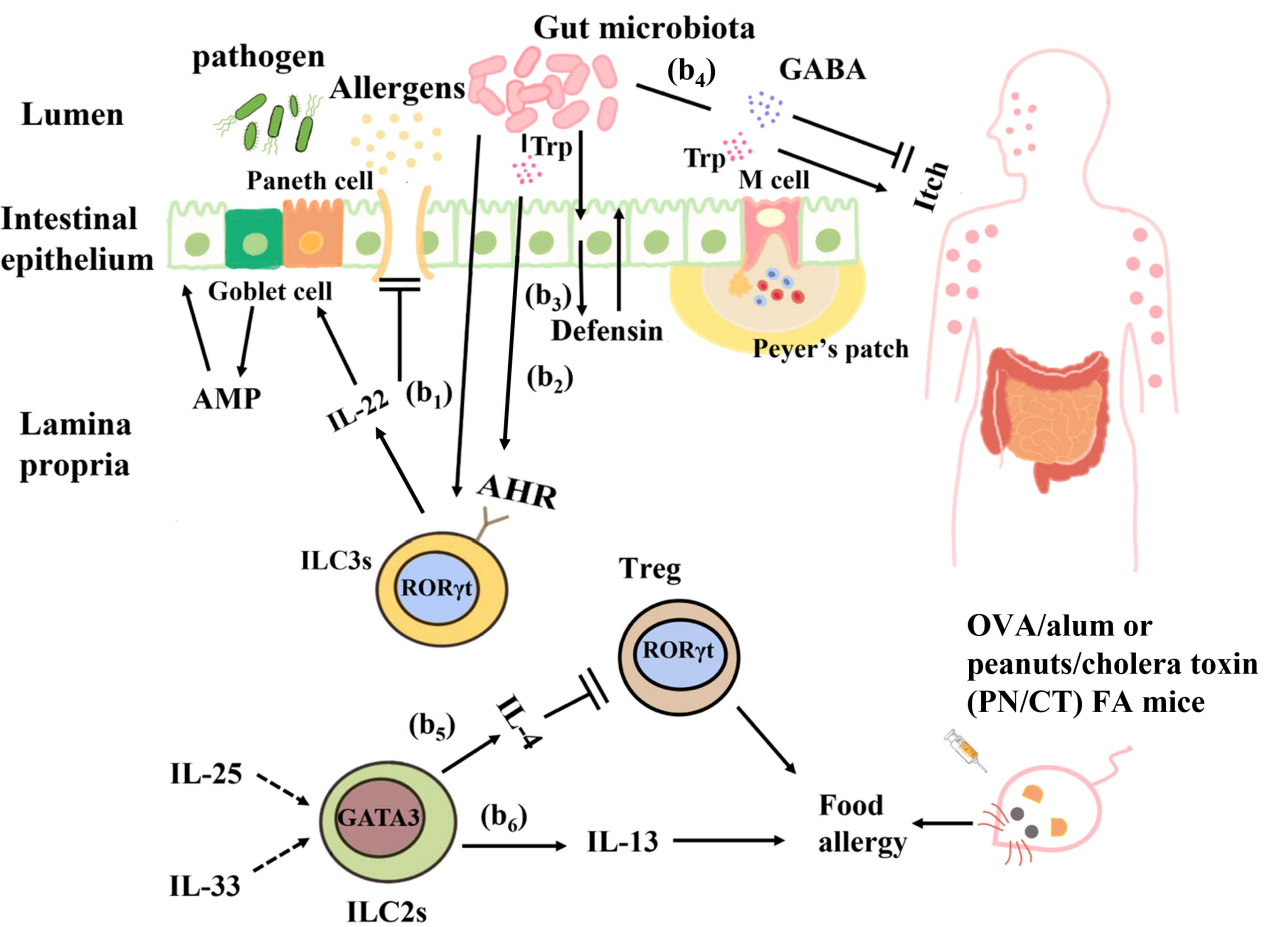
Interactions between the gut microbiota and ILCs in food allergy. (b1) *Clostridium* colonization causes ILC3s in the intestinal lamina propria to produce IL-22, which enhances epithelial barrier function by regulating goblet cells and Paneth cells to increase mucus and antimicrobial peptide production. (b2) Ligands produced from the dietary and bacterial-derived metabolite tryptophan bind to AHR on ILC3s and stimulate IL-22 production to regulate epithelial barrier permeability and prevent harmful substances from entering the blood circulation. (b3) The intestinal microbiota can directly interact with intestinal epithelial cells to produce defensins and strengthen their barrier function. (b4) Tryptophan produced by the gut microbiota causes pruritus, and γ-aminobutyric acid (GABA) produced by *Lactobacillus* and *Bifidobacterium* inhibits pruritus. (b5) In OVA/alum or peanuts/cholera toxin (PN/CT) FA mice, ILC2s inhibit antigen-specific Tregs by producing IL-4, thus aggravating allergic reactions. (b6) IL-33 and IL-25 elicit IL-13 production from ILC2s can induce mastocytosis and diarrhea symptoms in OVA/alum or PN/CT FA mice.

In OVA/alum or peanuts/cholera toxin (PN/CT) FA mice models, oral administration of OVA or peanuts can lead to type 2 inflammation, diarrhea and anaphylactic symptoms such as body temperature decrease *via* mast cell degranulation may appear. Research found when IL-25 and IL-33 which is involved in the activation of ILC2s are elevated, the number of intestinal ILC2s will be increased in the FA model mice, it indicates that ILC2s are involved in the pathogenesis of FA (222). Another research has reported that increased IL-25 can elicit IL-13 production from ILC2s *via* direct stimulation or indirect activation through IL-25 receptor-positive Th2 cells can induce mastocytosis and diarrhea symptoms (223). The skin barrier disruption could induce the activation of intestinal ILC2s *via* IL-33, which enhances mastocytosis and anaphylaxis in OVA-induced FA models (224). This suggests that adverse activation of ILC2s is involved in the allergic reaction. In addition, IL-4α knocked out PN/Ct-induced FA models of mice lacking an unrestricted form of the immunoreceptor tyrosine inhibitory motif (ITIM), in which ILC2s inhibits antigen-specific Tregs by producing IL-4. IgE reactivity of mast cells is further enhanced by the production of IL-4/IL-13, leading to exacerbation of anaphylaxis (225, 226). But there are other points of view as well, Chu et al. reported that ILC2s, based on the use of Thy1 neutralizing antibodies in PN/CT-induced FA mode, leads to FA-induced abdominal type 2 inflammation, but does not lead to IgE production, gastrointestinal symptoms, and allergic reactions (227). These differences may be due to different FA models and mouse strains used, or due to the influence of gut microbiota (**Figure 3**).

### 4.3 Colorectal cancer

**Figure 4:**
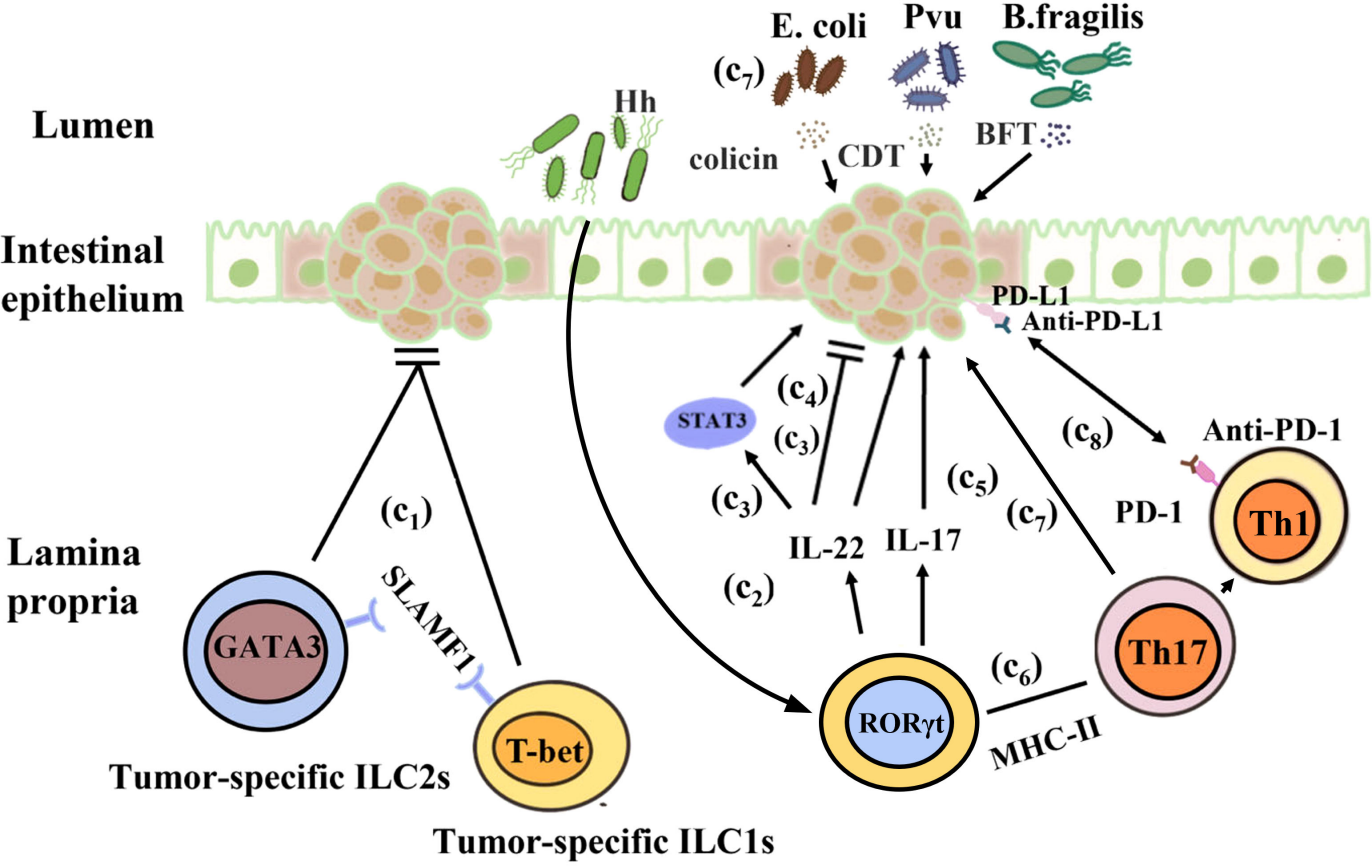
Interactions between the gut microbiota and ILCs in colorectal cancer. (c1) *H. hepaticus* stimulates ILC3s to produce IL-22, which promotes colorectal cancer in an induced tumor model. (c2) IL-22 stimulates STAT3 activation in intestinal epithelial cells to maintain tumor development. (c3) IL-22 controls colon tumorigenesis and the malignant proliferation of epithelial cells. (c4) IL-17 and IL-22 promote the development of invasive colon cancer. (c5) ILC3s reduce intestinal inflammation by inhibiting the activity of microbiota-specific Th17 cells in an MHC-II-dependent manner. (c6) In mice, ILC3-specific MHC-II supports anti-tumor Th1 cell immunity as well as anti-PD-1 immunotherapy. (c7) *Escherichia coli*, *Bacteroides fragilis*, ϵ-Proteus, and γ-Proteus can secrete colicin, BFT, and CDT, which are associated with colon cancer and can damage host DNA.

It has been suggested that there are complex interactions among ILCs, the microbiota, and cancer. The risk of cancer and tumorigenesis is linked to the increase in inflammatory mediators produced by ILCs (228). Under normal circumstances, IL-22 secreted by ILC3s can promote epithelial damage and repair. However, during tumor development, IL-22 is highly expressed and the expression of IL-22 binding protein (IL-22BP) is restricted, resulting in excessive production of IL-22 and promoting cancer occurrence. For example, a study showed that IL-22 is an anti-tumor cytokine that inhibits tumor growth in mice. Huber et al. found that IL22^-/-^ mice exhibit a susceptible to colon cancer, IL-22 plays a key role of in controlling tumorigenesis and epithelial cell proliferation in the colon. However, Patients with IBD have a considerably elevated risk of CRC, IL-22 can activate STAT3 in IECs to promote cell proliferation and play a major role in maintaining tumor development (229, 230). Another study showed that IL-22 promotes CRC development in a *Helicobacter hepaticus-*induced tumor model (230). In addition, a study showed that the reduction in IL-17 and IL-22 can prevent the development of invasive colon cancer in mouse models of inflammatory dysplasia (228). Furthermore, under pathological conditions, intestinal dysbiosis due to antibiotic or other reasons may cause ILCs release cytokines such as IL-17 and IL-22, which may lead to chronic inflammation and cancer. The reason maybe that IL-22 and IL-17 secreted by colon ILC3s are involved in the occurrence of inflammation and tumor growth. ILC3s express antigen presenting factor major histocompatibility complex-II (MHC-II), which reduces intestinal inflammation by limiting the activity of microbiota-specific Th17 cells in an MHC-II-dependent manner. The proportion of ILC3s decreases significantly in CRC, leading to an increase in the inflammatory activity of Th17 cells in the intestine. The composition of the intestinal microbiota is altered in mice lacking ILC3-specific MHC-II, that restricts Th1 cells and type 1 immunity in the intestine indirectly, leading to the further development of aggressive CRC, which does not respond to anti-tumor Th1 cells and or anti-PD-1 immunotherapy (231, 232). Cytokines secreted by ILCs can promote the proliferation of some bacteria, including *Enterococcus hirae*, *Barnesiella intestinihominis*, *Bacteroides fragilis*, *Bacteroides thetaiotaomicron*, *Bifidobacterium breve*, and *Bifidobacterium longum*, which have been proved to boost the therapeutic effect of cancer (233, 234). However, some microorganisms such as *Escherichia coli*, *Bacteroides fragilis*, *ϵ* and γ *proteobacteria* also have cancer-promoting effects. They can produce colicin, *Bacteroides fragilis* toxin, and lethal cell-tumescent toxins. These molecules have clinically and experimentally been linked to colon cancer (235, 236), and can directly or indirectly damage host DNA through the induction of reactive oxygen species (237, 238). Furthermore, ILCs may also limit the niche of commensal bacteria such as *Escherichia coli* and *Helicobacter pylori*, which have been demonstrated to facilitate cancer development (236, 239). Recently, Transcriptomic studies found that ILC1s, ILC3s, and ILC3/NKs exist in a healthy gut, but not ILC2s. Additional tumor-specific ILC1s-like and ILC2 subsets were identified in CRC patients. Signaling lymphocytic activation molecule family member 1 (SLAMF1) selectively expressed on tumor-specific ILCs, and it is an anti-tumor biomarker in CRC (240). In a steady-state environment, ILCs play an important role in regulating the intestinal immune environment and maintaining the balance between the tumor promotion and tumor inhibition effects of symbiotic bacteria (**Figure 4**).

### 4.4 Parasitic infection

The innate immune response is essential for the prompt control of parasitic infections and comprises a variety of innate immune cells, including ILCs. *Toxoplasma gondii* is a widespread protozoan whose sexual development occurs in the intestinal epithelium of the definitive host (241). This pathogen can cause the zoonotic disease toxoplasmosis, which can become chronic. In fact, chronic toxoplasmosis is one of the most common infections around the world (242). After oral infection in susceptible C57BL/6 mice, *T. gondii* invades IECs and the lamina propria, resulting in the production of abundant cytokines and Th1-mediated immune responses in the gut. This model can simulate IBD (242). Among all pro-inflammatory cytokines, IFN-γ plays the most prominent role in eliminating *T. gondii* infection, *T. gondii* activates the TLR-11 dependent signaling pathway through the MyD88 adapter protein and initiates the production of IL-12, which stimulates the expression of IFN-γ in ILC1s, and NK cells, the latter leads to cell starvation and limitations on the growth of *T. gondii*. Oral infection of wild-type mice with *T. gondii* results in a decrease in the number of ILC3s in the lamina propria and downregulation of RORγt expression. This is mainly because IL-12 signals downregulate RORγt expression, induce the conversion of ILC3s into IFN-γ-expressing ILC1s and promote IFN-γ production (71), thus contributing to the control of *Toxoplasma* infection (81). In a high-dose model of *T. gondii*, IL-23 induces local up-regulation of matrixmetalloproteinase-2 (MMP-2) through IL-22, which has pro-inflammatory effects and can further exacerbate intestinal inflammation (243). In addition, a recent study showed that ileitis caused by *Toxoplasma* infection is attenuated in IL-22-deficient mice (244). In humans, the sporozoites of *Plasmodium* are first injected into the blood of the host from the salivary glands of mosquitoes (245) and then infect the liver. In both human and mouse infection models, NK cells act as key immune cells for the early and sustained control of *Plasmodium* infection (246). This indicates that the role of IL-22 in inflammation may have tissue specificity, it can perform different functions based on its quantity and duration in the organization. For example, IL-22 may play a pathogenic role in keratinocytes and small intestinal epithelial cells, while playing a protective role in colon and rectum epithelial cells, lung epithelial cells, and liver cells. In the same time, variety of experimental models (pathogen or chemically induced) may also lead to different effects of IL-22.

**Figure 5:**
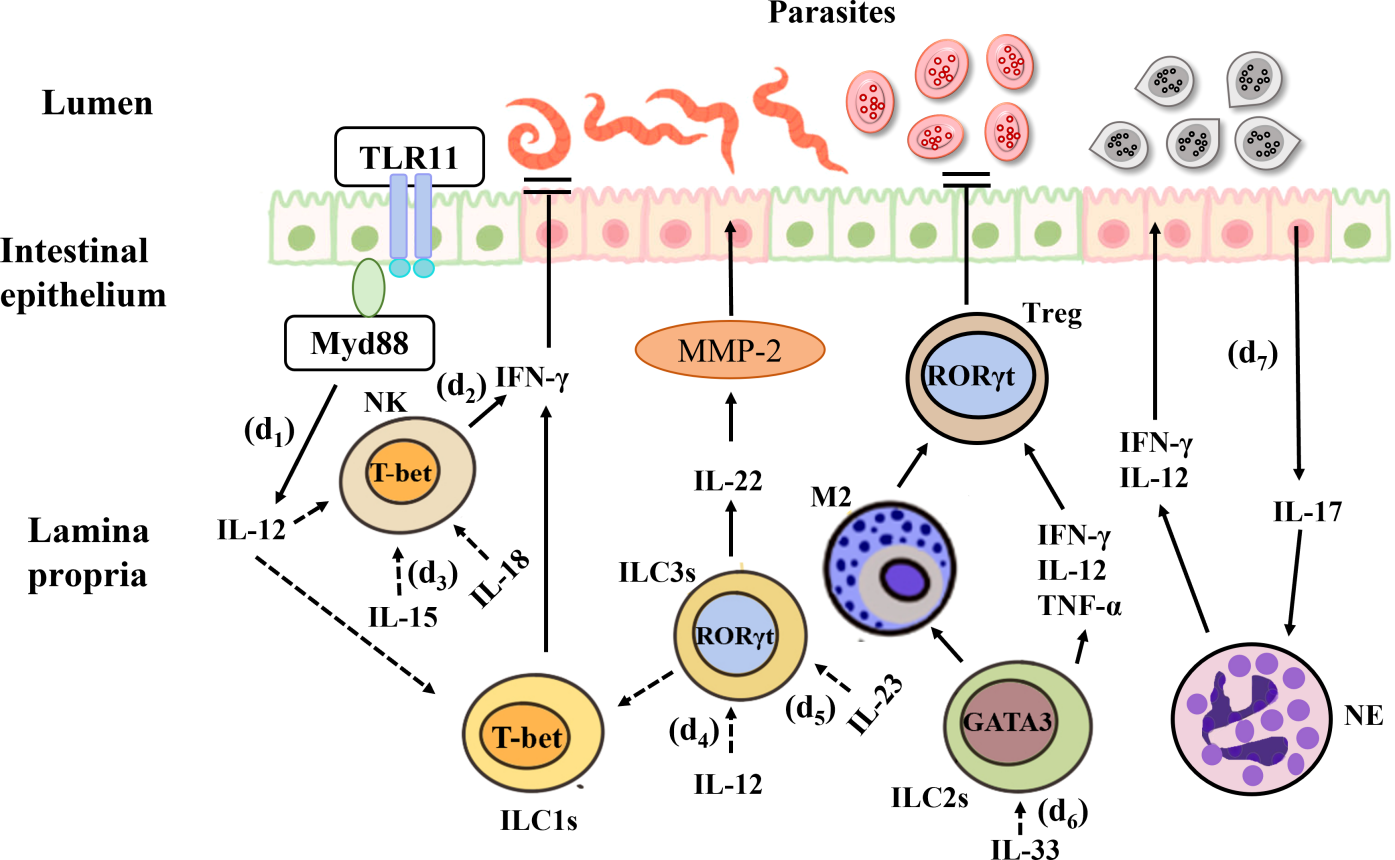
Interactions between the gut microbiota and ILCs in parasitic infection. (d1) *T. Gondii* activates the TLR-11 dependent signaling pathway through the MyD88 adapter protein and initiates the production of IL-12, which stimulates the expression of IFN-γ in ILC1s and NK cells, the latter limits the growth of *T. Gondii*. (d2) IL-15 enhances the expression of NK cells, and promotes greater killing potential, thereby clearing the *Cryptosporidium*. (d3) IL-12 signals downregulate RORγt expression, induce the conversion of ILC3s into IFN-γ-expressing ILC1s and promote IFN-γ production, thus contributing to the control of *Toxoplasma* infection. (d4) IL-23 induces up-regulation of matrixmetalloproteinase-2 (MMP-2) through IL-22, and can further exacerbate intestinal inflammation. (d5) IL-33 can drive the expansion of ILC2s and reduce the inflammatory mediator, and the production of anti-inflammatory M2 macrophages, which in turn amplifies Foxp3^+^Treg, thus preventing the development of ECM. (d6) During *Eimeria falciformis* infection, a large amount of IL-17 is produced in the chickens’ body, which may be achieved by recruiting concentrated granulocytes and inhibiting the expression of IL-12 and IFN-γ, and exacerbating inflammatory reactions.

In a study on experimental cerebral malaria (ECM) in C57BL/6 mice, it was shown that IL-33 can drive the expansion of ILC2s that produce type 2 cytokines (IL-4, IL-5, and IL-13) and reduce the inflammatory mediator IFN-γ, IL-12 and TNF-α, leading to the production of anti-inflammatory M2 macrophages. This, in turn, amplifies Foxp3^+^Treg, thus preventing the development of ECM (247). *Cryptosporidium* infection primarily occurs through the ingestion of oocysts in contaminated water (248). This parasite lives in IECs and can cause diarrhea in humans. Studies showed during infection, IL-15 activates peripheral blood mononuclear cells (PBMC), enhances the expression of NK cells, and promotes greater killing potential in both epithelial T cells and NK marker expressing cells, thereby clearing the *Cryptosporidium* (249). *Eimeria* spp. infection also occurs *via* the ingestion of fecal material containing parasite oocysts, causing severe inflammation in the intestinal mucosa (250). The research indicates that in the early stage of *Eimeria falciformis* infection, a large amount of IL-17 is produced in the body of chickens, which may be achieved by recruiting concentrated granulocytes and inhibiting the expression of IL-12 and IFN-γ (Th1 type cytokines) in order to promotes the occurrence and development of cecum lesions, and exacerbating inflammatory reactions (251). It suggests that ILC3s can respond to *Eimeria* infection. In addition, a study found that IFN-γ^−/−^ mice infected with *Eimeria* spp. experience greater weight loss and more severe intestinal histopathology following the depletion of IL-17A and IL-22. On the contrary, antibody neutralization has no significant effects in wild-type mice (252) (**Figure 5**).

## 5 Conclusion

In recent years, ILCs have become a research hotspot in the field of immunology and cell biology. Despite their small percentage, these lymphocytes provide protection against bacteria, parasites, and viruses, and also play a role in tissue repair and regeneration in the mucosa. However, ILCs are bidirectional, and their inflammatory effects on the body can aggravate intestinal inflammation and even cause cancer. ILCs are characterized by their ability to induce corresponding cytokines on the surface of the intestinal barrier to achieve rapid responses. Therefore, how the interaction between ILCs and the intestinal microbiota can be equilibrated and how internal homeostasis with the microbiota can be maintained to ensure that the cytokines secreted by ILCs play a protective role are worthy of further exploration. Nevertheless, some issues related to ILCs remain to be addressed. For example, whether the process of ILC trans-differentiation *in vivo* is reversible needs to be examined. Moreover, the differences between the subgroups produced during traditional developmental processes need to be clarified further. The two-way regulatory mechanism of ILCs in the body needs to be elucidated to explore the correlation between ILCs and various diseases and to provide new strategies for disease diagnosis and treatment. Therefore, comprehensive analysis methods are required to study the interaction between ILCs and the intestinal microbiota, and this could become a key topic for the treatment of clinical diseases in the future. Innovative ILC-based interventions should be explored to better understand the relationship between ILCs and diseases and achieve a more comprehensive understanding. This could help in safeguarding the health of the human race.

## Author contributions

YG did the literature review and wrote the main body of the article. YiL and ML co-supervised on finishing this review. YL, BR, ZL, XN constructed the idea.ML made valuable comments to the manuscript. All authors contributed to the article and approved the submitted version.
